# Outcomes of Surgical Tracheostomy on Mechanically Ventilated COVID-19 Patients Admitted to a Private Tertiary Hospital in Tanzania

**DOI:** 10.7759/cureus.32245

**Published:** 2022-12-06

**Authors:** Nadeem Kassam, Ally Zain, Sajida Panjwani, Salim Surani, Omar M Aziz, Kamran Hameed, Samina Somji, Hanifa Mbithe, Fatma Bakshi, Bonventura Mtega, Gloria Kinasa, Mariam Msimbe, Blessing Mathew, Eric Aghan, Harrison Chuwa, Christopher Mwansasu

**Affiliations:** 1 Internal Medicine, Aga Khan University Medical College, Dar es Salaam, TZA; 2 General Surgery, Aga Khan University Medical College, Dar es Salaam, TZA; 3 Medicine, Texas A & M University, College Station, USA; 4 Medicine, University of North Texas, Dallas, USA; 5 Internal Medicine, Pulmonary Associates, Corpus Christi, USA; 6 Clinical Medicine, University of Houston, Houston, USA; 7 Internal Medicine, Aga Khan Hospital, Dar es Salaam, TZA; 8 Anesthesiology, Aga Khan University Medical College, Dar es Salaam, TZA; 9 Anesthesia and Critical Care, Aga Khan University Medical College, Dar es Salaam, TZA; 10 Family Medicine, Aga Khan University Medical College, Dar es Salaam, TZA; 11 Medicine, Aga Khan University Medical College, Dar es Salaam, TZA; 12 Otolaryngology, Aga Khan University Medical College, Dar es Salaam, TZA

**Keywords:** surgical tracheostomy, tanzania, covid-19, tracheostomy outcomes, coronavirus

## Abstract

Objectives: The coronavirus disease 2019 (COVID-19) pandemic has resulted in an increase in the number of patients necessitating prolonged mechanical ventilation. Data on patients with COVID-19 undergoing tracheostomy indicating timing and outcomes are very limited. Our study illustrates­­­ outcomes for surgical tracheotomies performed on COVID-19 patients in Tanzania.

Methods: This was a retrospective observational study conducted at the Aga Khan Hospital in Dar es Salaam, Tanzania.

Results: Nineteen patients with COVID-19 underwent surgical tracheotomy between 16^th^ March and 31^st^ December 2021. All surgical tracheostomies were performed in the operating theatre. The average duration of intubation prior to tracheotomy and tracheostomy to ventilator liberation was 16 days and 27 days respectively. Only five patients were successfully liberated from the ventilator, decannulated, and discharged successfully.

Conclusions: This is the first and largest study describing tracheotomy outcomes in COVID-19 patients in Tanzania. Our results revealed a high mortality rate. Multicenter studies in the private and public sectors are needed in Tanzania to determine optimal timing, identification of patients, and risk factors predictive of improved outcomes.

## Introduction

As of 20th December 2021, the World health organization (WHO) officially reported over 270 million confirmed cases of coronavirus disease 2019 (COVID-19) globally, with approximately five million related deaths. This has resulted in increased Intensive Care Unit (ICU) admissions for respiratory failure and acute respiratory distress syndrome (ARDS) [1]. Our understanding of this disease, particularly in Sub-Saharan Africa, is still evolving; it has been observed that patients who suffer respiratory failure appear to be dependent on mechanical ventilation, posing an extra burden on healthcare systems [2]. Tracheostomies are normally performed when prolonged mechanical ventilation is anticipated in order to enable smooth weaning and reduce the length of ICU stay [3]. Generally, patients requiring a prolonged period of mechanical ventilation undergo tracheostomy typically after 10-21 days of endotracheal intubation [4,5]. Additionally, tracheostomies are also beneficial in reducing the work of breathing and improving patient comfort and communication by reducing the need for sedation [6]. Nonetheless, it is considered an aerosol-generating procedure that raises concerns when performed in COVID-19-infected patients [7]. Expert opinion on delayed tracheostomies is justified considering the reduced risk of transmission in the later course of illness [8,9]. Data on patients with COVID-19 undergoing tracheostomy indicating time and outcomes are very limited [10,11]. The few documented studies have yielded varying results with wide variations [10-12]. The dynamic nature of the pandemic, geographical variations, and limited data have been major obstacles to developing and reaching a consensus with regard to tracheostomy in COVID-19-infected patients, particularly in Sub-Saharan Africa.

This study primarily aimed to document the experience at our center, which may provide reference and aid to the growing body of knowledge about this disease.

## Materials and methods

This single-center retrospective study was conducted in the Intensive Care Unit (ICU) of the Aga Khan Hospital, Dar es Salaam, Tanzania after obtaining Intuitional Review Board (IRB) approval (approval #AKU/2022/024/fb/001). The Aga Khan Hospital is the largest private hospital in the country and the only Joint Commission International (JCI) accredited health facility in the region. All patients diagnosed with COVID-19 infections and who underwent surgical tracheostomy between 16th March 2020 and 31st December 2021 due to prolonged mechanical ventilation were included in the study. The Hospital follows the World Health Organization (WHO) living guidelines for COVID-19 and the latest guidelines for treatment for Adult Respiratory Distress Syndrome (ARDS) [13].

The ICU of the Aga Khan Hospital is capable of providing mechanical ventilation, hemodialysis, and inotropic support. Patients admitted to the ICU are reviewed daily by a multidisciplinary team that includes an intensivist, primary care physicians, residents from the department of internal medicine, physiotherapists, nutritionists, and when needed wound care nurses. The nursing team is set up to have a 1:1 ratio to provide maximum round-the-clock care and support, while physiotherapy is conducted 1-2 times daily and then individualized based on respiratory assessment. The decision for surgical tracheostomy is based on the consensus opinion of a multidisciplinary team. Patients who are intubated for more than 10 days are considered candidates. All tracheostomies were performed in the operating room via an open surgical approach whereby dissection to expose the trachea was carried out, followed by the opening of the trachea. A balloon-cuffed, non-fenestrated tracheostomy tube with a diameter ranging from 8 to 10 mm was inserted to maintain a closed circuit with minimal disconnections and suctioning to reduce aerosolization. Staff adhered to strict donning and doffing institutional protocols on adequate personal protective equipment (PPE). Staff in the operating room was limited to the anesthesiologist, surgeon, assistant surgeon, scrub nurse, and circulating nurse. Anticoagulants were stopped 12 hours prior and reinitiated within 24 hours post-procedure. The plan of care post tracheostomy was dependent on the need of a particular patient. Goals to decrease sedation, ventilator support, and trials of weaning were standardized. Shorter-acting analgesics were preferred over continuous infusion. ARDS protocol was deployed accordingly, and the goal was to convert to the lowest level of pressure support. Ventilator settings were adjusted continuously according to the metabolic and hemodynamic profile of patients. The trial of tolerating unassisted breathing was subjective with the aim of liberation from mechanical ventilation. Once achieved, patients were disconnected from the ventilator and were evaluated daily by the physiotherapist for participation in chest physiotherapy, range of motion, and trial of ambulation. The hospital provided family and social support with daily meetings with the next of kin as well as video calling to aid rehabilitation and motivation throughout the stay. 

Demographics, past medical history, available clinical information on admission, duration of mechanical ventilation, liberation from ventilation, and outcomes were retrieved from institutional medical records. Liberation of mechanical ventilation was defined as the first full 24-hour period without ventilator assistance. The Data was collected by research assistants who had experience working in the ICU and was verified by the Primary investigator for accuracy and completeness. Data were cleaned and explored before further analysis using RStudio (R Studio 2021.09.2, https://cran.rstudio.com/). The categorical data were summarized using frequency and percentage, while continuous variables were expressed as median with respective interquartile range (IQR). Laboratory parameters were compared on admission and before tracheostomy using Wilcoxon signed ranked sum test, and p< 0.05 was set as the significance level. All data derived were presented as tables and graphs.

## Results

A total of 19 patients were studied. The median age of our cohort was 62 years (IQR 50- 67), with the majority of them being male (n=13, 68%) of African origin (n=11, 58%), having a BMI of above >30 kg/m^2^ (n=9, 47%). More than one comorbid condition was entered whenever present; the majority of our participants were either diabetic (n=3, 16%), hypertensive (n=4, 21%), or both (n=8, 42%). Table 1 below illustrates the demographic characteristics of our study population. 

**Table 1:** Demographic of the study population ^1^;Median (IQR); n (%). DM; diabetic Mellitus, HTN; hypertension, BMI; Body mass Index, HIV; Human immunodeficiency virus

Characteristic	N = 19^1^
AGE	62 (50, 67)
SEX	
Female	6 (32%)
Male	13 (68%)
ETHNICITY	
African	11 (58%)
Asian	6 (31%)
Chinese	1 (5)
Others	1 (5)
BMI (Kg/m^2^)	29.8 (25.3, 33.6)
18.5 to < 25.0	3 (16%)
25.0 to 30.0	7 (37%)
> 30.0	9 (47%)
COMORBID	
None	2 (11%)
DM	3 (16%)
HTN	4 (21%)
DM, HTN	8 (42%)
HIV	2 (11%)
PREGNANT	1 (5%)
LOCATION PRIOR TO ADMISSION	
Home	11 (58%)
Referral	8 (42%)

Majority of the patients who underwent tracheostomy presented with difficulty in breathing and fever (n=9, 47.4%), presenting to the emergency department within seven days of symptoms (n=12, 63%). Most patients presented with a critical form of the disease (n= 18, 95%) requiring a non-re-breather mask (n=13, 68%) on admission to attain adequate oxygenation, as seen in Table 2 below.

**Table 2:** Clinical symptomatology of the cohort ^1^n (%), HFNC: High flow nasal cannula

Characteristic	N = 19^1^
PRESENTING SYMPTOMS	
Difficulty in breathing and fever	9 (47.4%)
Difficulty in breathing and fatigue	6 (31.5%)
Difficulty in breathing and cough	4 (21%)
DURATION OF SYMPTOMS	
< 7 days	12 (63%)
7+ days	7 (37%)
SEVERITY ON ADMISSION	
Severe	1 (5.3%)
Critical	18 (95%)
INITIAL OXYGEN SUPPORT AT EMERGENCY	
Non-rebreather mask	13 (68%)
HFNC	5 (26%)
Nasal prongs	1 (5.3%)

Table 3 below provides a comparison of laboratory parameters on initial presentation and before tracheostomy. Statistical significance was noted in the platelet count and CRP (C-Reactive Protein). 

**Table 3:** Comparative table of laboratory parameters ^1^;Median (IQR), PCT; CRP; C-reactive protein, WBC; White blood cell Procalcitonin, LDH; lactate dehydrogenase,

Characteristic		Before Tracheostomy, N = 19^1^	On Admission, N = 19^1^	p-value
WBC	10.8 ( 7.5- 15.5)	11.7 (9.4- 14.6)	9.5 (6.2- 15.4)	0.3
PLATELET	244 ( 179 -311)	184 (140- 291)	251 (242- 318)	0.036
PCT	0.45 (0.19- 1.15)	0.42 (0.16- 1.15)	0.47 (0.22- 0.97)	0.6
CRP	65 (14- 173)	20 (3- 65)	174 (65- 258)	<0.001
LDH	680 (531- 926)	615 (443- 885)	692 (565- 929)	0.4
FERRITIN	1,571 (825- 2,420)	1,926 (810- 2,420)	1,441 (993- 2,160)	>0.9
D-DIMER	3.26 (1.14- 5.27)	3.60 (2.67- 5.81)	2.06 (0.88- 4.36)	0.070

Table 4 illustrates the complete physiological profile and other ventilation parameters of our cohort.

**Table 4:** Parameters before tracheostomy PEEP; Positive End-Expiratory Pressure

Characteristics	N = 19^1^
MEDICATIONS PATIENTS RECEIVED	
Dexamethasone, Remdesivir and Tociluzimab	9 (47%)
Dexamethasone and Remdesivir	5 (26%)
Dexamethasone and Tociluzimab	3 (16%)
Dexamethasone	2 (11%)
PEEP BEFORE TRACHEOSTOMY (CMH_2_O)	
PEEP of < 5	0 (0%)
PEEP of 5-8	7 (37%)
PEEP of > 8	12 (63%)
MEAN PaO_2_/FiO_2 _BEFORE TRACHEOSTOMY	65 (SD:27; min=32, max=143)
< 100	17 (89%)
100 to <200	2 (11%)
200-300	0 (0%)
MEAN PaO_2_/FiO_2_ AFTER TRACHEOSTOMY	115 (SD: 86, min=30,max=293)
< 100	12 (63%)
100 to <200	3 (16%)
200-300	4 (21%)
TRACHEOSTOMY INTERVALS	
Average number of days from 1^st^ Intubation to tracheostomy	16 SD: 5; (min=10, max=25)
Average number of days from Tracheostomy to ventilator liberation	27 SD: 43; (min =3, max=105)
REINTUBATION	2 (11%)
ORGAN SUPPORT DURING ADMISSION	
Inotropes and Dialysis	5 (26%)
Inotropes	14 (74%)

**Figure 1:**
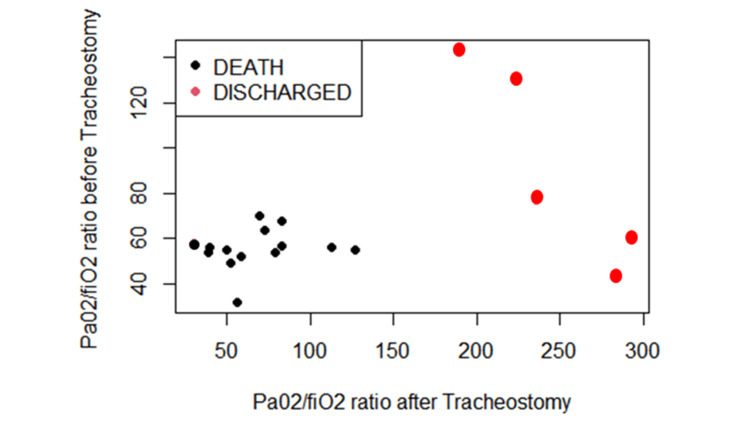
PaO2/FiO2 before and after tracheostomy.

Figure 1 below is a scattered plot diagram that highlights the relationship of PaO2/FiO2 before and after tracheostomy. Only five (26.3%) of the 19 patients were liberated from mechanical ventilation and were eventually decannulated and discharged. All five patients exhibited improved PaO2/FiO2 after tracheostomy. 

**Figure 2:**
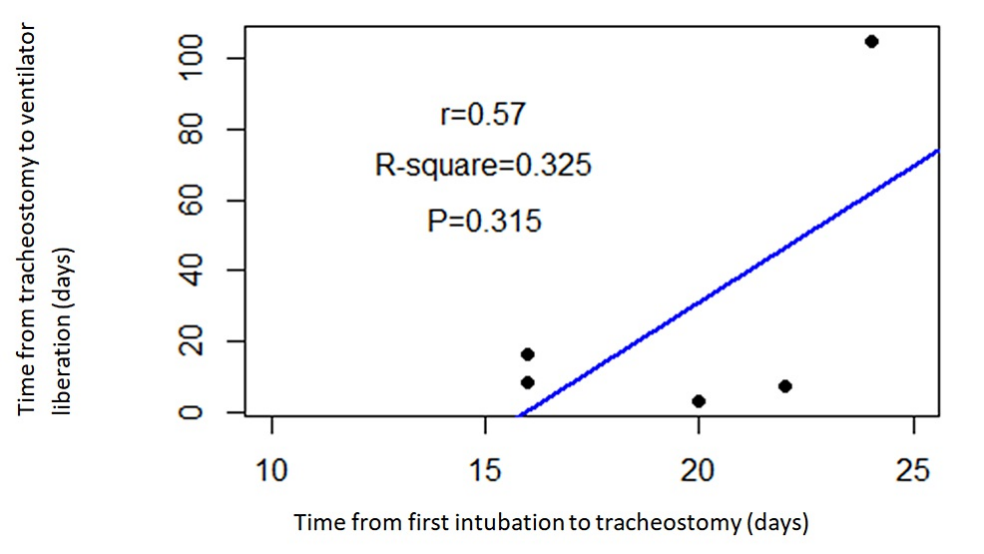
Correlation between time from first intubation to tracheostomy (X-Axis) and time from tracheostomy to ventilator liberation (Y-Axis)

The mean time of intubation to tracheostomy and tracheostomy to ventilator liberations was 16 days (min 10, max 25) and 27 days (min 3, max 105), respectively. There was a moderate positive correlation as seen in Figure 2 below between the time from intubation to tracheostomy and the time from tracheostomy to ventilator liberation (r=0.57, P= 0.315). 

**Figure 3:**
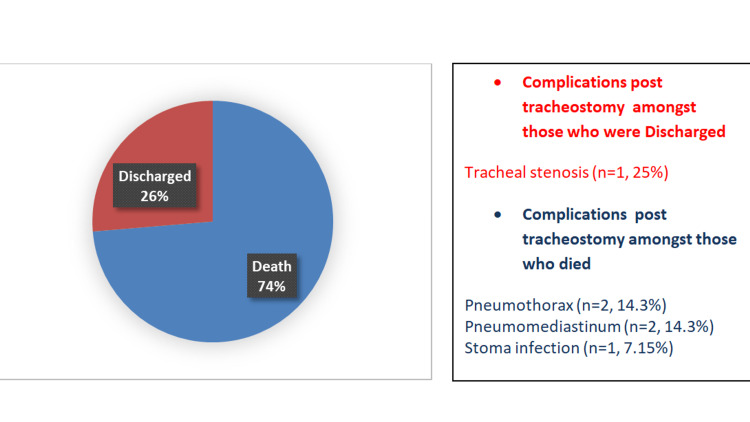
illustration of outcomes and complications post-surgical tracheotomy

More than half (n=14, 73.6%) of the patients who underwent surgical tracheostomy due to ventilator dependency did not make it to hospital discharge, as seen in Figure 3 below.

## Discussion

To our knowledge, this is the first study in Sub-Saharan Africa to document outcomes of surgical tracheostomy in critically ill COVID-19 patients dependent on mechanical ventilation. Our results highlight that only five were liberated from mechanical ventilation, decannulated, and successfully discharged among the 19 patients who underwent surgical tracheostomy. Our findings reflect significantly higher mortality rates and lower ventilator liberation rates compared to data published globally [14,15]. Our study cannot attribute factors associated with higher mortality rates amongst our cohort due to the small sample size. Single-center studies have indicated the presence of sepsis [16] to be a major risk factor associated with higher mortality rates. Factors associated with a high mortality rate among critical COVID-19 patients, such as advanced age, the burden of obesity, diabetes mellitus, and hypertension, as well as a critical form of illness on presentation [17-20], were all observed among our study population.

Additionally, the majority of our patients who underwent surgical tracheostomy were suffering from a severe form of ARDS (PaO2/FiO2 <100) requiring higher PEEP (Positive End-Expiratory Pressure) values (>8 cmH2O) for adequate oxygenation. PaO2/FiO2 is the most important oxygenation index in COVID-19 patients. A lower value reflects hypoxemic respiratory failure and is considered an independent predictor of mortality [21]. Patients requiring higher PEEP tend to have fewer ventilator-free days and are likely to suffer from acute kidney injury necessitating renal replacement therapy [22], as also observed in our cohort.

The timing of tracheotomy in COVID-19 patients is still debatable, and contrasting results have been published. Findings from single-center studies revealed; early tracheostomies [23-25] decrease the duration of mechanical ventilation and increase ventilator liberation rates by facilitating a faster weaning process and decreasing the length of hospitalization. Nevertheless, some centers tend to postpone tracheostomies as much as possible due to the high risk of aerosolization. The consensus from the meta-analysis found no difference in mortality and de-cannulation rate between early and late tracheostomy [14,15]. Surgical tracheostomies at our center were generally done after 10 days of ventilator dependence. The average number of days from tracheostomies to ventilator liberation among those who survived was 27 days. Our results are similar to studies done in Italy [26] and New York [27]. Bedside tracheostomy is also a successful alternative and an accepted procedure for COVID -19 patients [28], provided it is done in an airborne isolation room with appropriate personal protective equipment (PPE). Despite bedside tracheostomy gaining popularity, there are no clinical trials comparing both techniques in COVID-19 patients [29]. Thus, the choice between the two depends largely on hospital resources [30], intuitional expertise [31], and patient-related factors [32]. It was well observed that pneumothorax and pneumomediastinum were the main complications observed in our cohort. This may be a result of a false tract during placement, accidental perforation [33] or because of COVID-19 itself.

Our study had various limitations. Our sample size was too small to draw any comparison. Secondly, our center is a private setup and thus is not a direct reflection of the Tanzanian population. The study design did not allow us to follow up on long-term outcomes, even if it is beyond the scope of the study. Like any other single-center study, it lacks the scientific objective to support widespread change in medical practice. 

## Conclusions

This study is one of the first to describe outcomes for patients who underwent surgical tracheostomy due to COVID-19 in Tanzania. Despite our results illustrating a high mortality rate, the five patients who survived hospital discharge all exhibited improved oxygenation as reflected by their PaO2/FiO2 ratio. We observed very few complications secondary to tracheostomies amongst our study population. Multicenter studies in the private and public sectors are needed in Tanzania to determine the statistical significance of the results. Furthermore, periodic updating of practice and protocols is needed to identify methods and timing of tracheostomies.
